# Supplementary material for: Structure and rational engineering of the PglX methyltransferase and specificity factor for BREX phage defence
Source: Nat Commun. 2024 Aug 22;15:7236. doi: 10.1038/s41467-024-51629-7 (PMC11341690; doi:10.1038/s41467-024-51629-7)
Supplement: Supplementary file 1 — Supplementary Information [file 41467_2024_51629_MOESM1_ESM.pdf]

Supplementary Materials

Structure and rational engineering of the PglX methyltransferase and specificity factor for BREX phage defence

Sam C. Went[a], David M. Picton[a], Richard D. Morgan[b], Andrew Nelson[c], Aisling Brady[d], Giuseppina Mariano[e], David T. F. Dryden[a], Darren L. Smith[c], Nicolas Wenner[d], Jay C. D. Hinton[d], Tim R. Blower[a,*]

[a]Department of Biosciences, Durham University, South Road, Durham, DH1 3LE, UK.

[b]New England Biolabs, 240 County Road, Ipswich, MA 01938, USA.

[c]Faculty of Health and Life Sciences, Northumbria University, Newcastle Upon Tyne, NE1 8ST, UK.

[d]Institute of Infection, Veterinary and Ecological Sciences, University of Liverpool, Liverpool, L69 7ZB, UK.

[e]Department of Microbial Sciences, Faculty of Health and Medical Sciences, University of Surrey, Guildford, GU2 7XH, UK.

[*]To whom correspondence may be addressed. Email: timothy.blower@durham.ac.uk, tel: +44(0)1913343923.

Keywords: BREX, phage defence, PglX, methyltransferase, Ocr

Supplementary Figures

Supplementary Figure 1

Defense system	Percentage
DMS_other	55.87
BrxU	15.98
RM_type_IIG	9.78
RM_type_HNH	6.52
Paris	2.21
RM_type_I	1.95
RM_type_IV	1.47
Mokosh_TypeII	0.89
PsyrTA	0.89
Juk	0.53
septu_type_I	0.53
cbass_type_I	0.42
AbiE	0.32
SoFic	0.21
DRT_class_I	0.16
dXTPase	0.16
Mokosh_TypeI	0.16
argonaute_solo	0.11
cas_type_other	0.11
DRT_class_III	0.11
gabija	0.11
qatABCD_other	0.11
retron_IV	0.11
RosmerTA	0.11
SEFIR	0.11
shedu	0.11
zorya_type_II	0.11
3HP	0.05
AbiL	0.05
AbiU	0.05
AbiH	0.05
AVAST_type_II	0.05
DRT_class_II	0.05
GAO_19	0.05
hachiman_type_I	0.05
Menshen	0.05
Menshen_other	0.05
PifA	0.05
PT_DndABCDE	0.05
pycsar_unknown	0.05
RM_type_II	0.05
ShosTA	0.05
viperin_solo	0.05
RloC	0.05

Supplementary Figure 1. Co-occurrence of defense systems between *pglX* and *pglZ* of BREX. Of 1902 defense genes identified between *pglX* and *pglZ*, the largest identified group was GmrSD-family Type IV restriction enzyme BrxU (15.98%), whilst PARIS was found in 2.21% of cases. DMS_other are systems that PADLOC cannot categorically identify but contain a component of a known defence system, making up 55.87%, ~1062, of the total systems.

Supplementary Figure 2

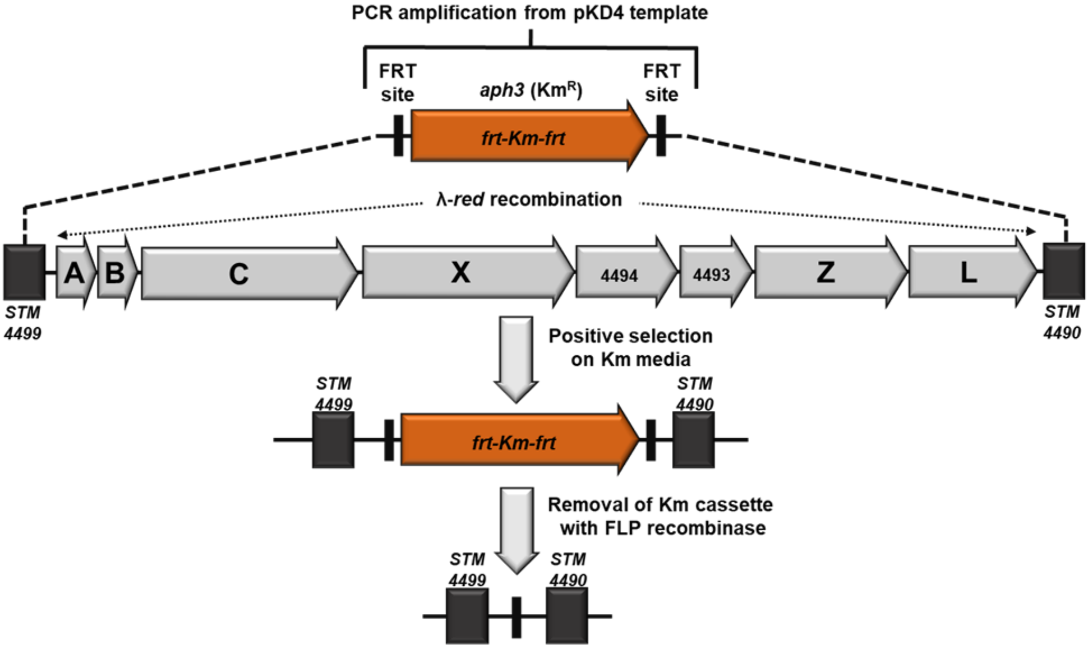

Supplementary Figure 2. Generation of *S.* Typhimurium D23580 BREX phage defence island knockout using Lambda red recombination. All genetic material between *STM4498* and *STM4491* (inclusive) was removed, and the kanamycin resistance cassette was cured.

Supplementary Figure 3

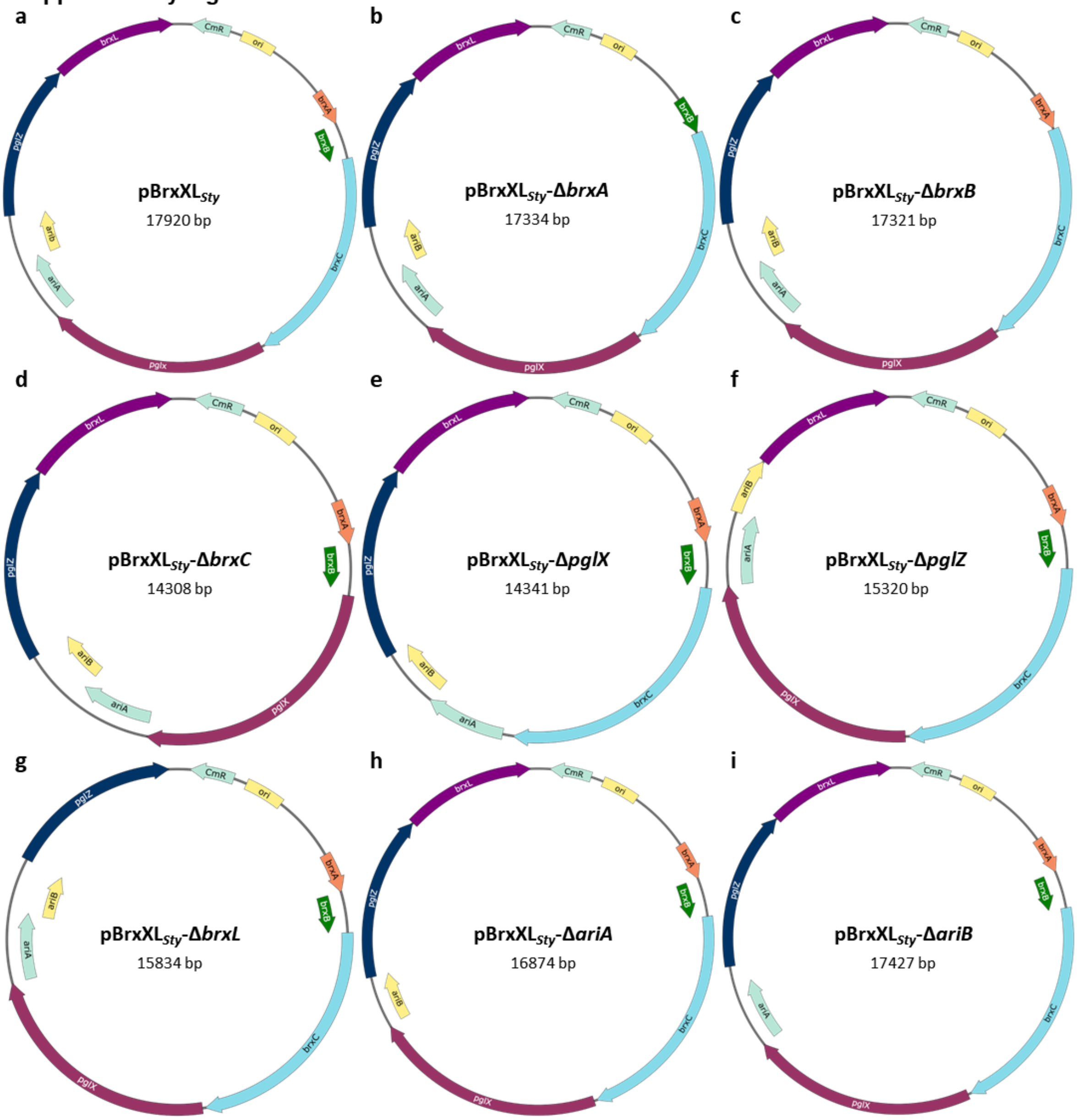

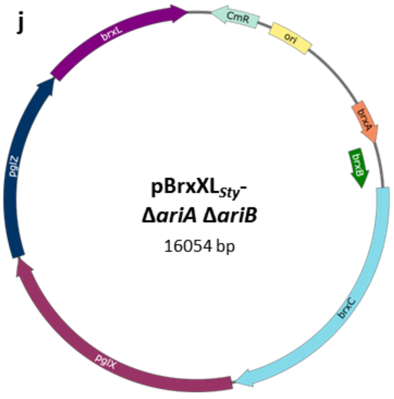

Supplementary Figure 3. ***Salmonella*** **D23580 BREX plasmids produced for this study**. The initial pGGA-based pBrxXL$_{Sty}$ plasmid (**a**) was previously produced by golden gate assembly[33] and used as a template for the production of gene knockout derivatives (**b-j**) by Gibson assembly.

Supplementary Figure 4

a

Phage	BREX sites	pBrxXL$_{Sty}$	pBrxXL$_{Sty}$-Δ*brxL*	pBrxXL$_{Sty}$-Δ*ariA*Δ*ariB*
TB34	120	0.03 ± 0.02	7.50 x10^{-6} ± 3.46 x10^{-6}	0.01 ± 2.96 x10^{-3}
Trib	0	1.19 ± 0.11	0.70 ± 0.27	0.94 ± 0.23
Baz	0	1.35 ± 0.46	1.34 ± 0.81	2.08 ± 1.67
Alma	110	0.03 ± 3.21 x10^{-3}	2.7 x10^{-4} ± 1.61 x10^{-4}	6.35 x 10^{-3} ± 4.39 x10^{-3}
Pau	84	0.19 ± 0.13	3.73 x10^{-4} ± 2.65 x 10^{-4}	0.14 ± 0.27
BB1	51	0.11 ± 0.05	0.27 ± 0.91	<4.29 x10^{-8} ± 1.61 x10^{-8}
Jura	0	2.58 ± 0.11	1.95 ± 1.37	1.82 ± 0.53
Mak	1	0.78 ± 0.10	3.77 ± 2.90	0.67 ± 0.12
Bam	1	1.06 ± 0.60	1.71 ± 0.69	0.59 ± 0.16
CS16	13	0.03 ± 0.02	0.01 ± 2.39 x10^{-3}	0.06 ± 0.02
Mav	13	0.15 ± 0.02	0.06 ± 4.08 x10^{-2}	0.09 ± 0.10
Sip	84	0.04 ± 0.01	2.19 x10^{-5} ± 2.57 x10^{-4}	5.81 x10^{-3} ± 2.06 x10^{-2}

EOP ≥ 10^{-1} | 10^{-1} > EOP ≥ 10^{-2} | 10^{-2} > EOP ≥ 10^{-3} | 10^{-3} > EOP ≥ 10^{-4} | 10^{-4} > EOP

b

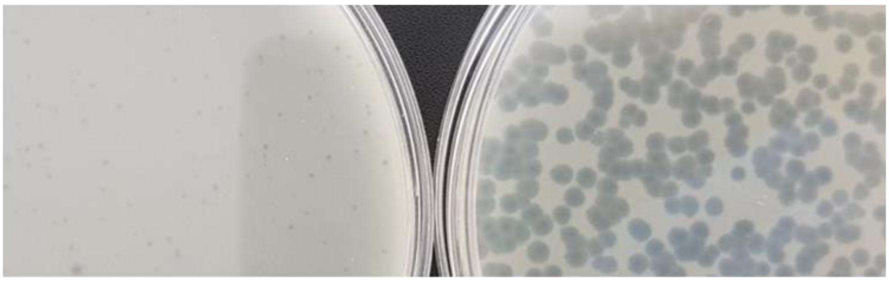

10^{-4} dilution of phage Alma, on *E. coli* DH5α pBrxXL$_{Sty}$

10^{-6} dilution of phage Alma, on *E. coli* DH5α pTRB507

c

Phage	EOP WT/ΔBREX	EOP Δ*brxL*/ΔBREX
SCW1	3.33 x 10^{-4} ± 1.43 x 10^{-4}	7.09 x 10^{-4} ± 1.16 x 10^{-4}
SCW2	0.93 ± 0.35	0.29 ± 0.08
SCW3	0.18 ± 0.09	0.16 ± 0.02
SCW4	1.15 ± 0.11	0.74 ± 0.26
SCW5	0.84 ± 0.23	1.10 ± 0.24

EOP ≥ 5 x 10^{-1}

5 x 10^{-1} > EOP ≥ 10^{-1}

10^{-1} > EOP ≥ 10^{-3}

EOP < 10^{-3}

Supplementary Figure 4. Wild type and mutant BREX loci show diverse defence. (**a**) EOPs of phages were tested against *E. coli* DH5α pBrxXL$_{Sty}$ plasmids with *E. coli* DH5α pTRB507 as control. Values are mean EOPs from triplicate data, shown with standard deviation. (**b**) Representative plates for EOP assays with phage Alma, using *E. coli* DH5α pBrxXL$_{Sty}$ against *E. coli* DH5α pTRB507 as control. (**c**) Efficiency of Plating (EOP) for *Salmonella* phages tested on *Salmonella* D23850Δφ against a control of *Salmonella* D23580ΔφΔBREX, and on *Salmonella* D23850ΔφΔ*brxL* against a control of *Salmonella* D23580ΔφΔBREX. Values are mean EOPs from triplicate data, shown with standard deviation. The EOP data for WT/ΔBREX are the same as shown in Fig. 1b, reproduced here for ease of comparison.

Supplementary Figure 5

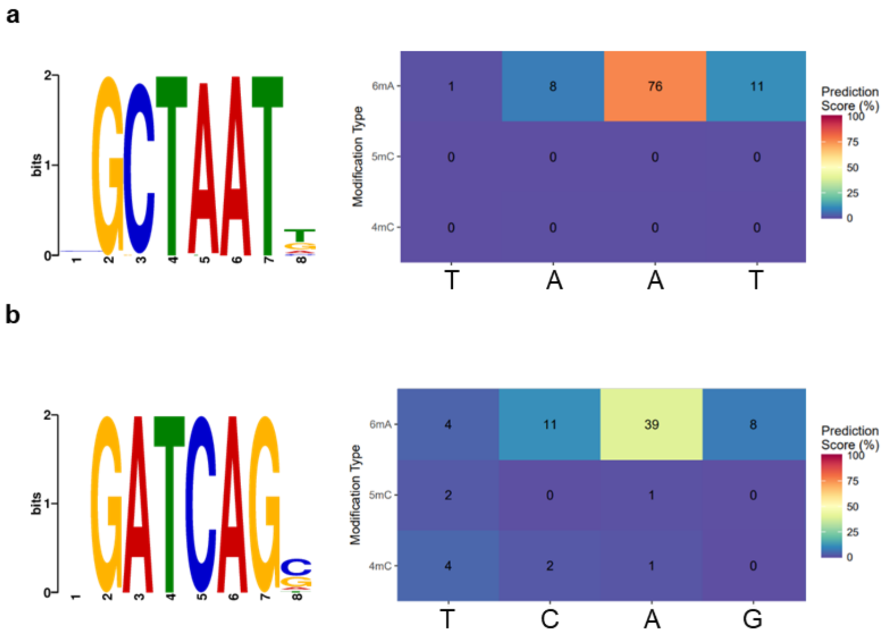

Supplementary Figure 5. Methylation analysis detected BREX motifs. Tombo (left) and nanodisco (right) analysis of genomic methylation from (**a**) *Escherichia fergusonii* and (**b**) *Salmonella* D23580 BREX systems. Tombo results represent enriched motifs around detected methylation signals, as identified by MEME (https://meme-suite.org/meme/). Nanodisco results show the type and position of DNA modification within DNA motifs identified by Tombo.

Supplementary Figure 6

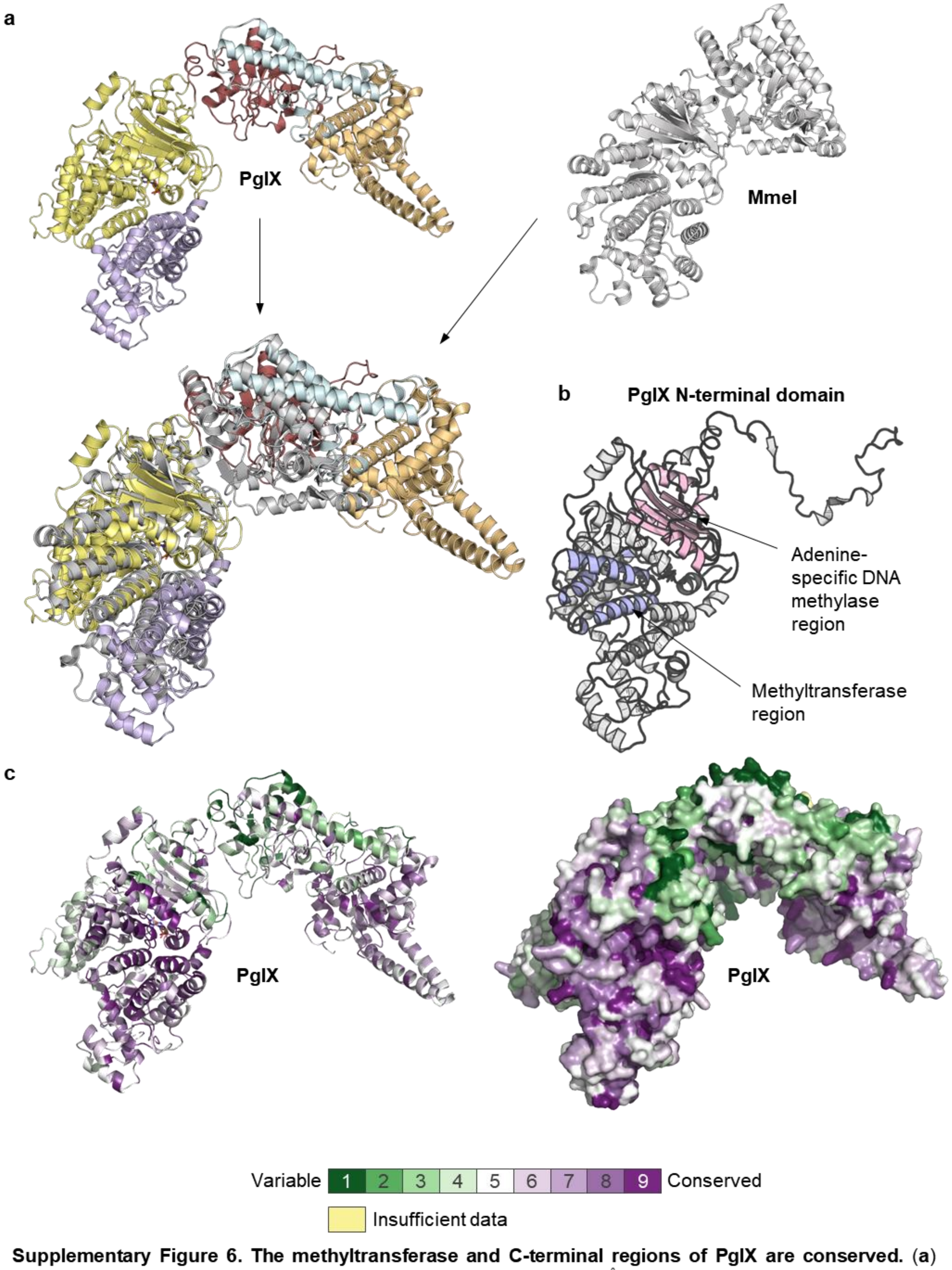

Supplementary Figure 6. The methyltransferase and C-terminal regions of PglX are conserved. (**a**) superposition of MmeI shown in gray (5HR4) onto PglX (RMSD = 7.1 Å). (**b**) Position of the predicted MmeI-like DNA methyltransferase region (light blue) and adenine-specific DNA methylase region (light pink) as positioned within the N-terminal domain (gray) of PglX. (**c**) Ribbon and surface views of conserved residues in PglX, as calculated by ConSurf[43].

Supplementary Figure 7

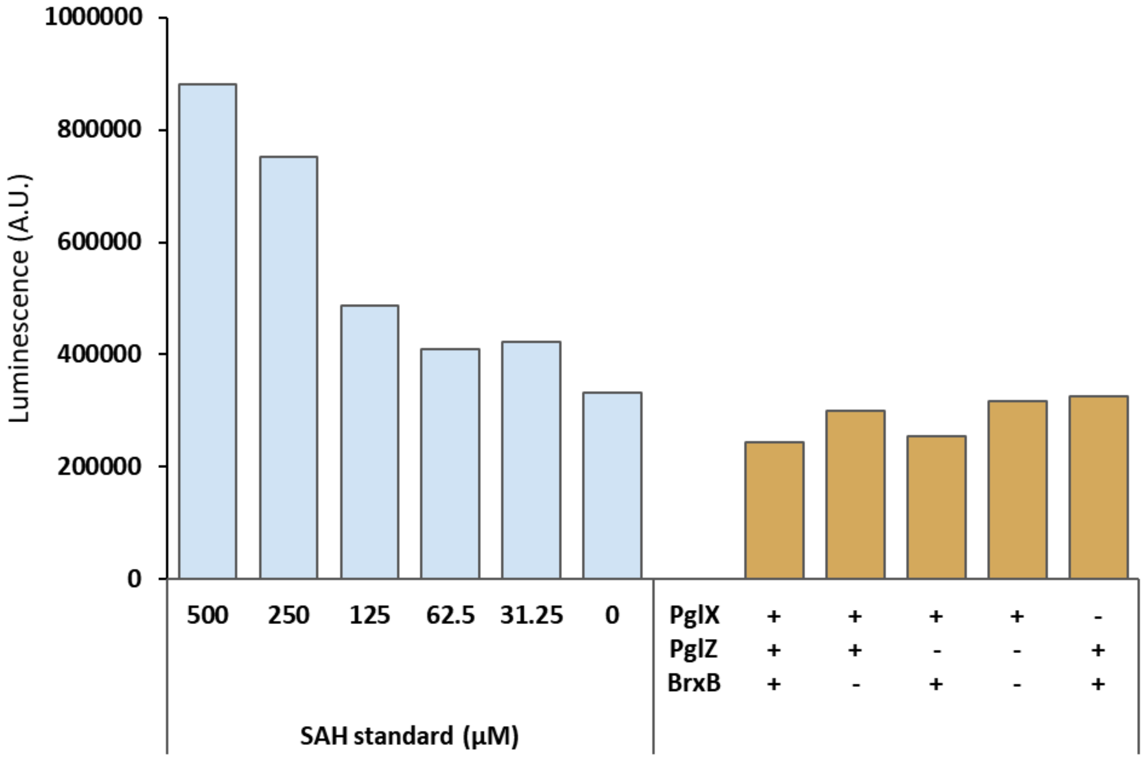

Supplementary Figure 7. *In vitro* methyltransferase activity analysis of purified PglX shows no methyltransferase activity. Activity of PglX alone or in combination with PglZ and BrxB, relative to SAH positive controls measured indirectly through production of the SAH methylation reaction byproduct using the MTase-Glo kit (Promega). Data are representative of two replicates.

Supplementary Figure 8

Strain	EOP
pBrxXL$_{Sty}$-*ΔpglX*	5.68 x10^{-1} ± 3.94 x10^{-1}
pBrxXL$_{Sty}$-Δ*pglX* pBAD30-*ocr*	1.22 ± 0.8
pBrxXL$_{Sty}$-Δ*pglX* pBAD30-*gp5*	1.06 ± 0.35

Supplementary Figure 8. Ocr and Gp5 do not activate the *Salmonella* PARIS system. EOPs of TB34 tested against *E. coli* DH5α pBrxXL$_{Sty}$-*ΔpglX* co-expressing either Ocr or its *Salmonella* homologue, Gp5, with *E. coli* DH5α pTRB507 as control. Values are mean EOPs from triplicate data, shown with standard deviation.

Supplementary Figure 9

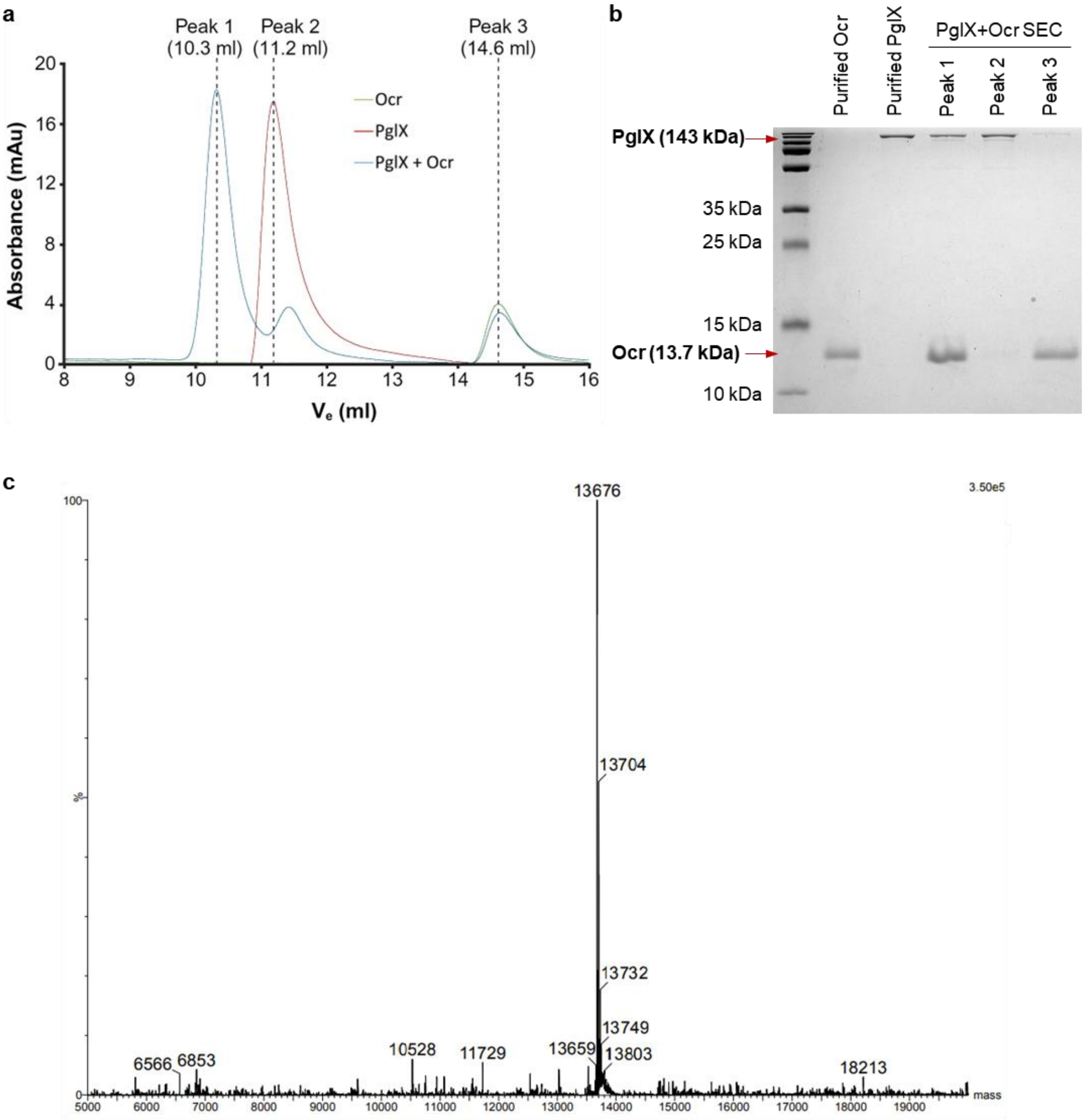

Supplementary Figure 9. PglX directly interacts with Ocr *in vitro*. (**a**) Analytical size exclusion chromatography of purified PglX, purified Ocr, and a mixed sample of PglX pre-incubated with Ocr. Elution volumes (V_e) of significant peaks are labelled. Traces show absorbance at 280 nm. (**b**) SDS-PAGE analysis of purified Ocr (lane 2), purified PglX (lane 3) and peak fractions from the mixed PglX and Ocr sample shown in (a). (**c**) Mass spectrometry results for purified Ocr.

Supplementary Figure 10

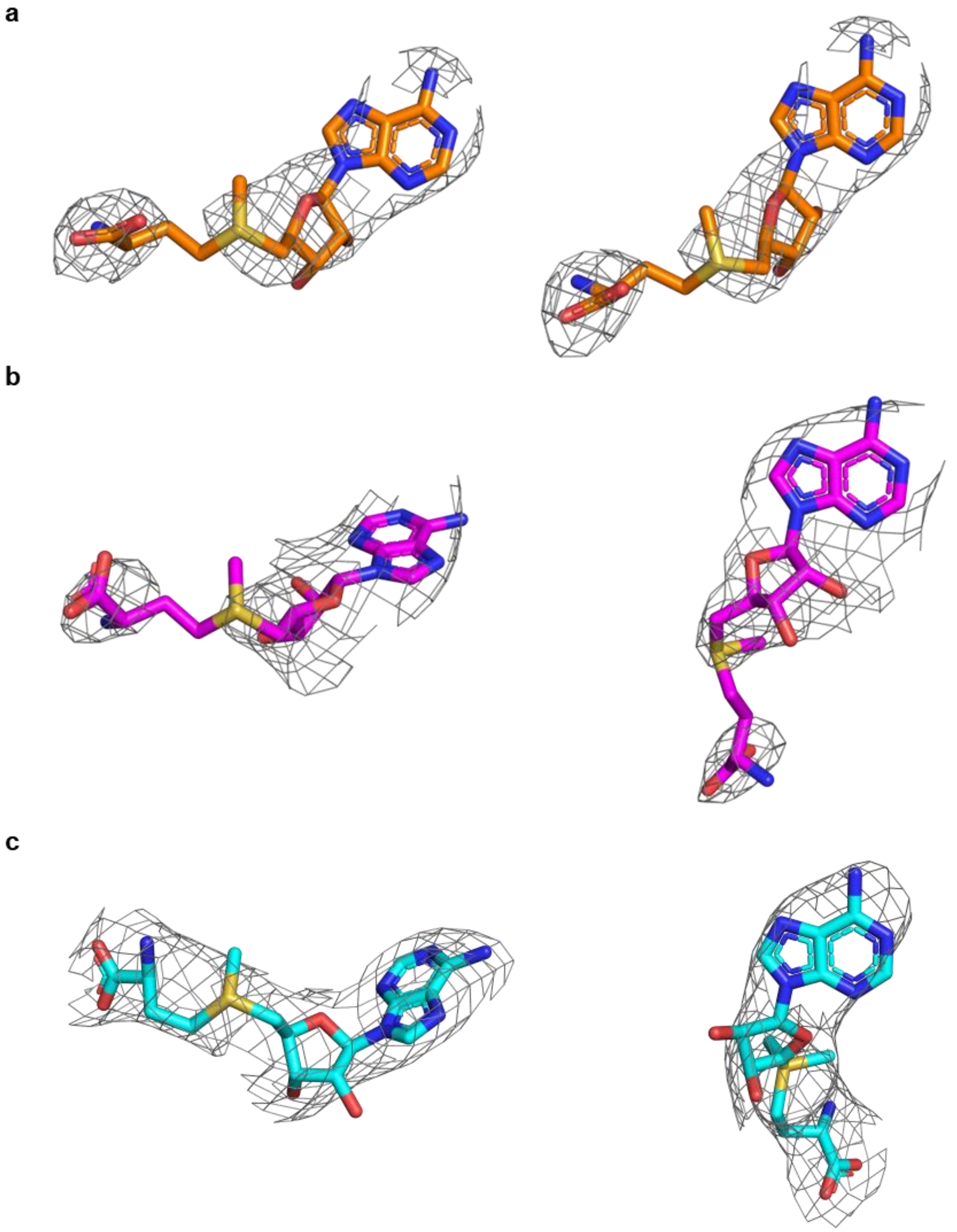

Supplementary Figure 10. Poses of SAM ligands differ at 3.5 Å resolution. Stick representation of SAM ligands, bound to (**a**) chain A and (**b**) chain B of the PglX-SAM structure; and (**c**) chain A of the PglX-SAM:Ocr structure. The displayed F_0-F_c electron density omit maps generated using Polder are contoured to 3σ. Ligands are colored orange, magenta and cyan for (a), (b) and (c), respectively. Left-hand – ligand oriented by position of sulphur group; right-hand – ligand oriented by position of adenine. Whilst the tail is held in the same location within each PglX protomer, there is substantial flexibility of pose provided at this resolution.

Supplementary Figure 11

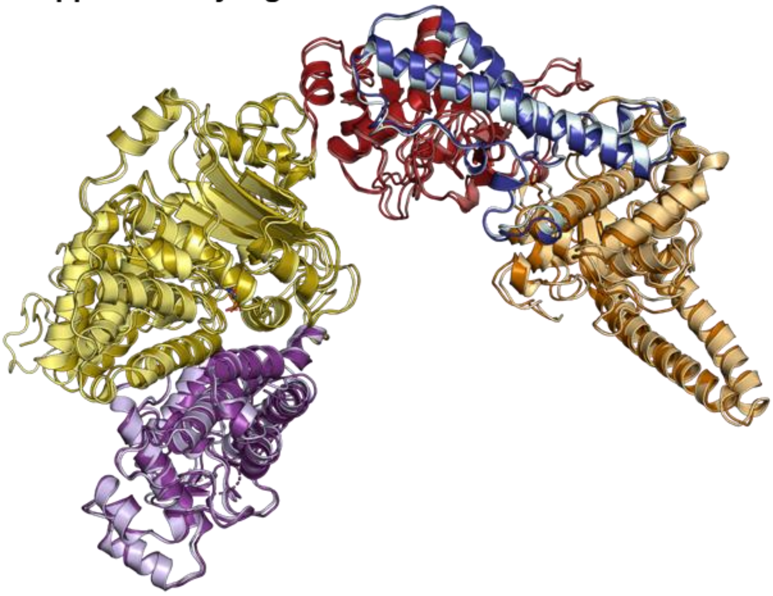

Supplementary Figure 11. Superposition of a protomer of PglX from the PglX-SAM structure (lighter shades), with PglX from the heterotetrameric PglX-SAM:Ocr complex (darker shades; RMSD = 1.34). Colored as per **Fig. 4**.

Supplementary Figure 12

a

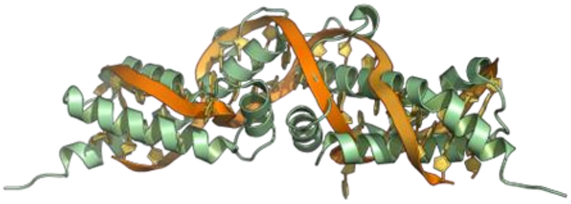

b

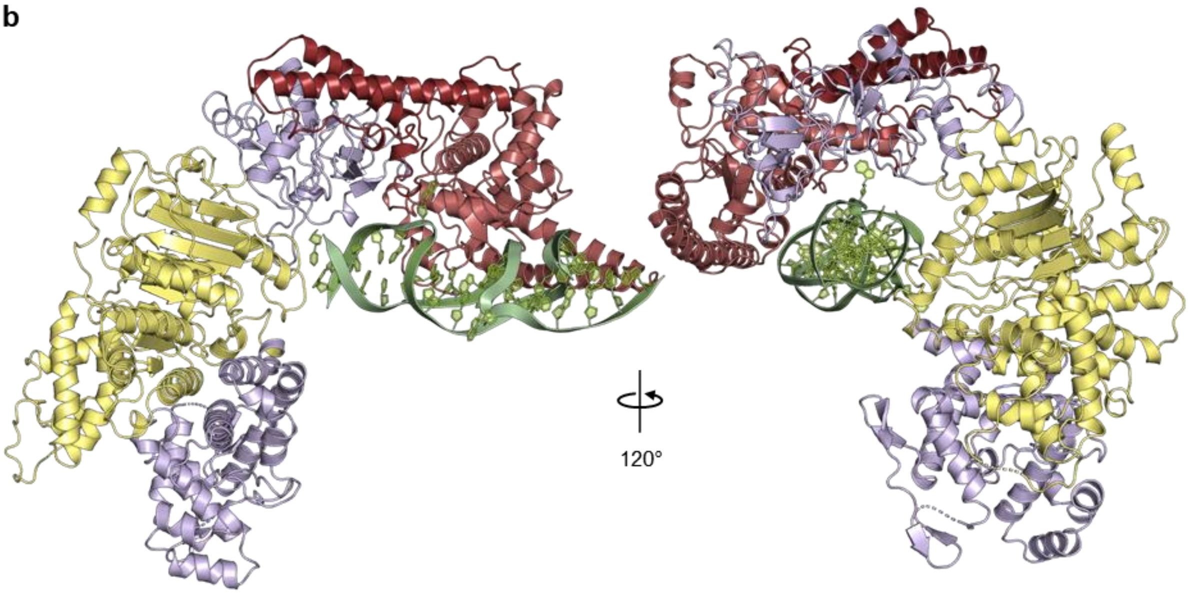

Supplementary Figure 12. Ocr binding suggests that PglX binds DNA along the inside surface of the C-terminal domain. (**a**) Alignment of an Ocr dimer (2Y7C) with a model of representative B-form DNA molecule (2Y7H). (**b**) Superposition of the model DNA molecule from (a) onto the Ocr molecule in the PglX-SAM:Ocr complex.

Supplementary Figure 13

a

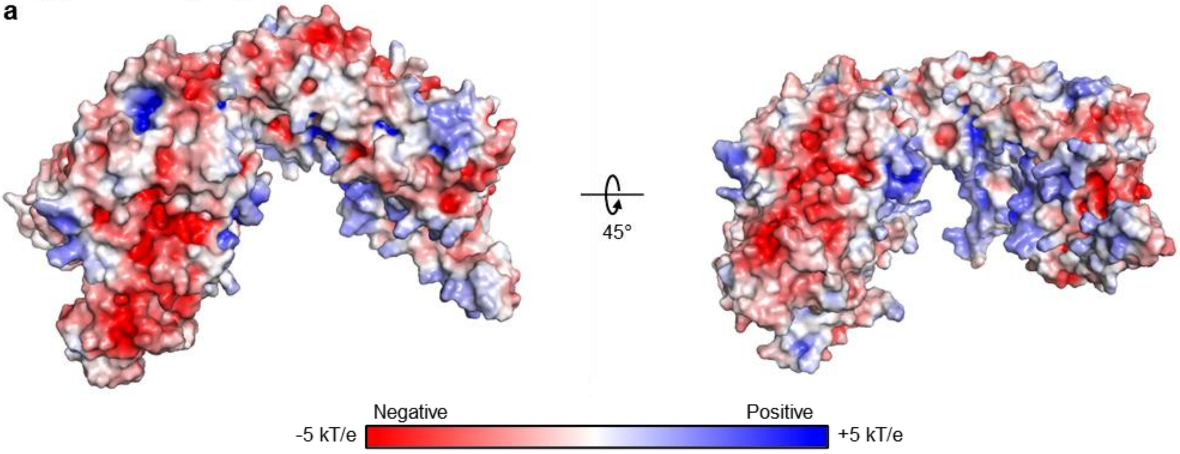

b

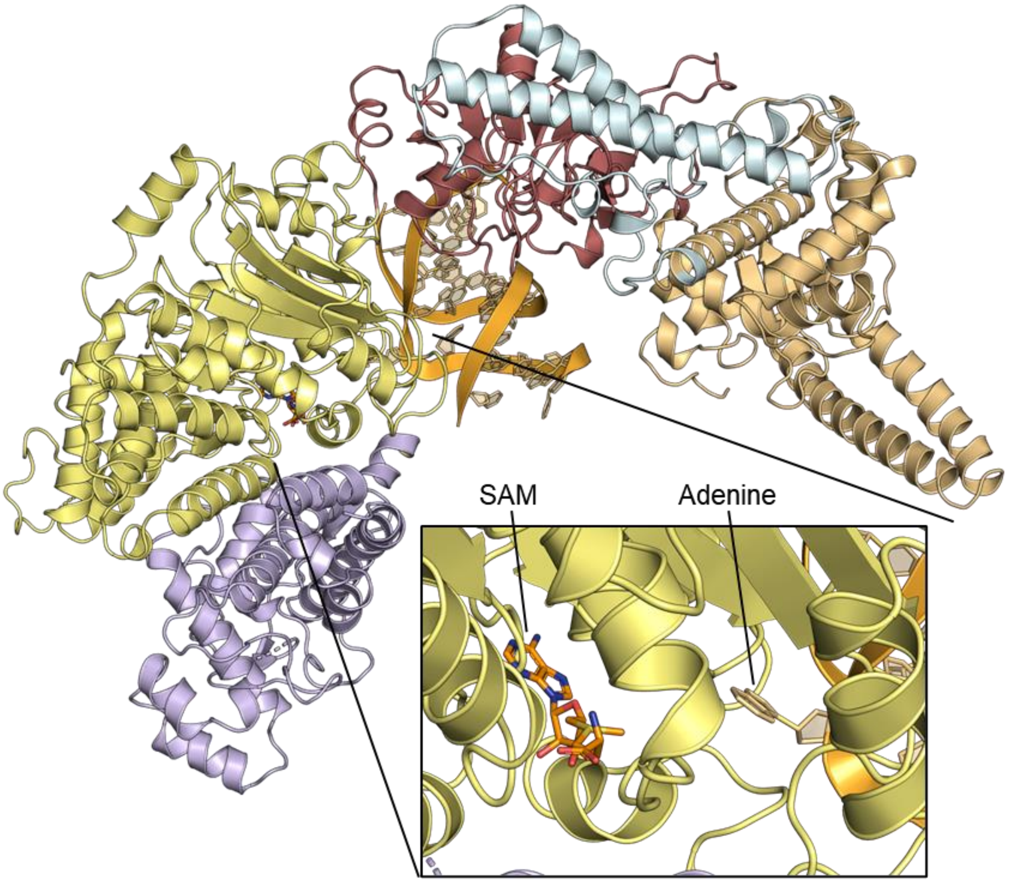

Supplementary Figure 13. PglX displays a negatively charged surface within the predicted DNA binding region. (**a**) Surface charge of PglX, showing a long negatively charged surface area along the C-terminal region into the target recognition domain. (**b**) Superposition of the 13 bp model DNA molecule from the structure of MmeI (5HR4) onto the solved structure of PglX. Inset; the position of the flipped-out adenine base in the MmeI model DNA molecule relative to the SAM molecule in PglX.

Supplementary Figure 14

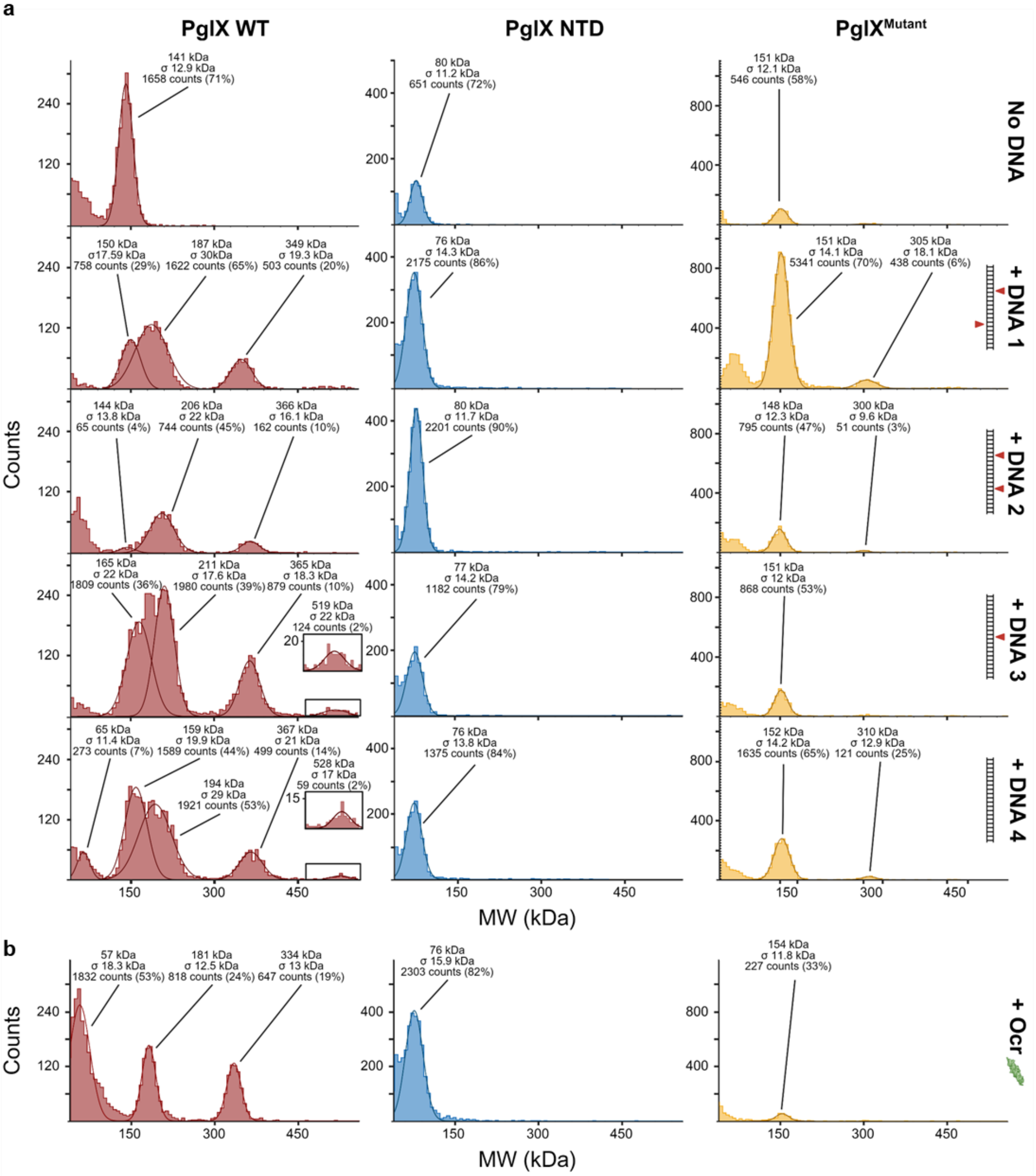

Supplementary Figure 14. Raw counts and peak statistics for PglX and PglX mutants binding to DNA and Ocr. (**a**) Mass photometry (Refeyn) assays examining the binding of PglX WT (red), PglX NTD only (blue) and PglX^{Mutant} (yellow), in the absence or presence of 120 bp dsDNA substrates. DNA 1, two BREX motifs, one on each strand; DNA 2, two BREX motifs, both on same strand; DNA 3, on BREX motif; DNA 4, no BREX motifs. Insets show larger complexes formed with DNA 3 and DNA 4 for PglX WT. (**b**) Mass photometry of proteins in (a), binding to Ocr (green). Selected peaks are labelled with mean molecular weight, standard deviation of gaussian fits, total counts per peak and percentage of counts per peak relative to total counts for each run. Data are representative of three independent experiments.

Supplementary Figure 15

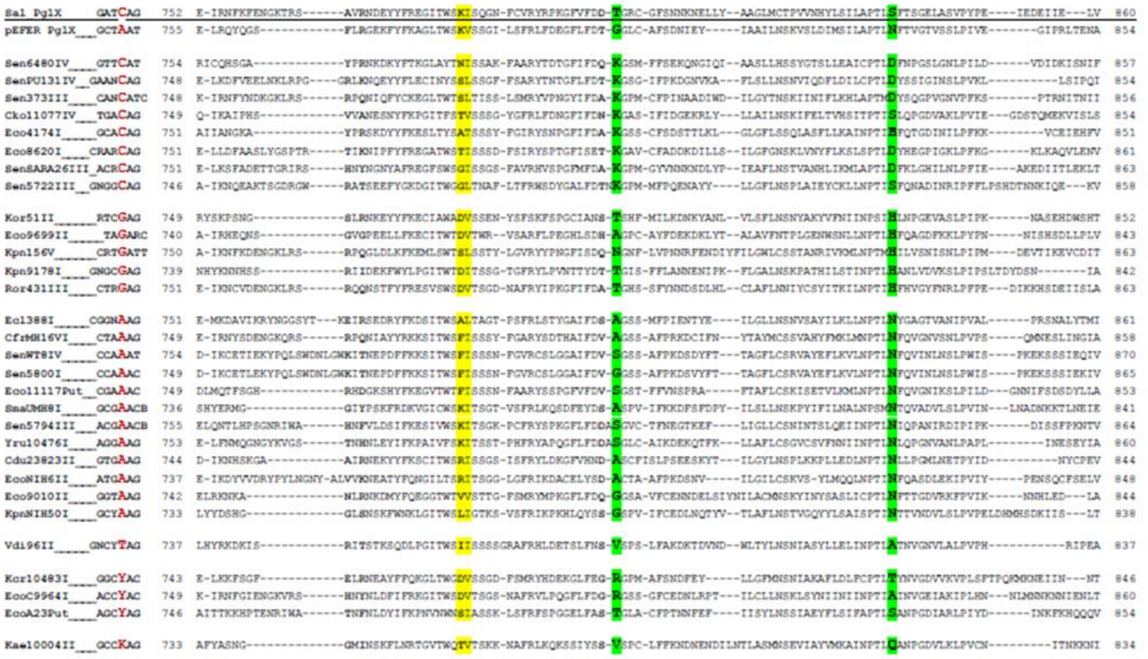

Supplementary Figure 15. Example of the PglX alignments used for design of PglX mutants. The residues in PglX which aligned with residues in MmeI controlling target sequence recognition were identified. The sequence of PglX was then aligned with homologues with known or predicted BREX recognition motifs and covariance between target residues and DNA recognition were identified. The above alignment represents identification of covariance of position -1 of the *Salmonella* BREX recognition motif (relative to the modified adenine) at PglX residues T802 and S838 (highlighted in green).

Supplementary Tables

Supplementary Table 1. PglX mutants designed to alter DNA motif recognition.

Mutant number	Motif position[1]	Current motif base	Mutations	Predicted change	Predicted motif
1	-1	C	T802T; S838H	G	GATGAG
2			T802N; S838H	G	GATGAG
3			T802A; S838N	A	GATAAG
4			T802G; S838N	A	GATAAG
5			T802V; S838A	T	GATTAG
6	-2	T	K782F; D801V	A	GAACAG
7			K782R; D801D	G	GAGCAG
8			K782R; D801S	G	GAGCAG
9			K782D; D801S	C	GACCAG
10			K782A; D801S	N	GANCAG
11	-3	A	A684L; S687R	C	GCTCAG
12			A684G; S687K	C	GCTCAG
13			A684K; S687D	G	GGTCAG
14			A684R; S687A	G	GGTCAG
15			A684H; S687S	G	GGTCAG
16			A684V; S687Q	T	GTTCAG
17			A684T; S687Q	T	GTTCAG
18	-4	G	A766R; R768Q	C	CATCAG
19			A766R; R768D	C	CATCAG
20			A766H; R768F	A	AATCAG
21			A766T; R768H	A	AATCAG
22			A766V; R768A	T/N	T(N)ATCAG
23	+1	G	Swap entire loop with pEFER PglX loop (AAs 591 - 600)	T	GATCAT

[1] Relative to modified adenine base.

Supplementary Table 2. Bacterial strains and bacteriophages used in this study.

Bacterial Strains

Strain	Genotype	Source
Escherichia coli		
DH5α	F- Φ80lacZΔM15 Δ(lacZYA-argF) U169 recA1 endA1 hsdR17 (rk-, mk+) phoA supE44 λ-thi1 gyrA96 relA1	Invitrogen
ER2796	K-12 F λ- fhuA2 Δ(lacZ)r1 glnV44 mcr-62 trp-31 dcm-6-zed-501::Tn10hisG1 argG6 rpsL104 dam-16::Kan xyl-7 mtlA2 metB1 (mcrB-hsd-mrr) 144::IS10	NEB
BL21 (DE3)	B; F^- *ompT* gal dcm lon $hsdS_B(r_B{}^- m_B{}^-)$ λ(DE3 [lacI lacUV5-T7p07 ind1 sam7 nin5]) $[malB^+]_{K-12}(\lambda^S)$	Invitrogen
Salmonella		
D23850Δϕ		(Owen *et. al.*, 2017)
D23850Δϕ pSIM5-tet	Temperature inducible λ red recombineering plasmid, temperature sensitive replication, tetracycline resistant (TcR)	(Owen *et. al.*, 2017)
D23850ΔϕΔBREX		(Rodwell *et al.*, 2021)
D23850ΔϕΔ*brxL*		This study

Bacteriophages

Phage	Host	Genbank accession	Source
TB34	*E. coli*	OX001802.1	Environmental isolate (Kelly et. al., 2023)
Trib	*E. coli*	OX016465.1	Environmental isolate (Kelly et. al., 2023)
Baz	*E. coli*	LR880803.1	Environmental isolate (Kelly et. al., 2023)
Alma	*E. coli*	OV101294.1	Environmental isolate (Kelly et. al., 2023)
Pau	*E. coli*	LR865361.1	Environmental isolate (Kelly et. al., 2023)
BB1	*E. coli*	MT843274.1	Environmental isolate (Kelly et. al., 2023)
Jura	*E. coli*	LR999871.1	Environmental isolate (Kelly et. al., 2023)
Mak	*E. coli*	OX001577.1	Environmental isolate (Kelly et. al., 2023)
Bam	*E. coli*	OW991346.1	Environmental isolate (Kelly et. al., 2023)

CS16	*E. coli*	LR999870.1	Environmental isolate (Kelly et. al., 2023)
Mav	*E. coli*	LR990702.1	Environmental isolate (Kelly et. al., 2023)
Sip	*E. coli*	OU734268.1	Environmental isolate (Kelly et. al., 2023)
T7	*E. coli*	V01146.1	Lab strain (ATCC)
SCW1	*Salmonella*	None	Environmental isolate, this study.
SCW2	*Salmonella*	None	Environmental isolate, this study.
SCW3	*Salmonella*	None	Environmental isolate, this study.
SCW4	*Salmonella*	None	Environmental isolate, this study.
SCW5	*Salmonella*	None	Environmental isolate, this study.

Supplementary Table 3. Primers used in this study.

Primer	Sequence	Notes
**Gibson assembly primers for creation of pBrxXL$_{Sty}$ knockouts (KO)**		
TRB1734	CGATGAAAACGTTTCAGTTTGCTCATGGAAAACGGTGTAA	pBrxXL$_{Sty}$ *pglX* Gibson assembly KO FWD (1)
TRB1735	CGCAGGCGATCGTCGAAGCTTTACTGAAGACGAATCCGGT	pBrxXL$_{Sty}$ *pglX* Gibson assembly KO REV (1)
TRB1736	ACCGGATTCGTCTTCAGTAAAGCTTCGACGATCGCCTGCG	pBrxXL$_{Sty}$ *pglX* Gibson assembly KO FWD (2)
TRB1737	TTACACCGTTTTCCATGAGCAAACTGAAACGTTTTCATCGCTCTGGA	pBrxXL$_{Sty}$ *pglX* Gibson assembly REV (2)
TRB1738	ATGCACCGGAGATTATTTAAATCTGGAATGAAATGGGATT	pBrxXL$_{Sty}$ PARIS Gibson assembly FWD (1)
TRB1739	TTACACCGTTTTCCATGAGCAAACTGAAACGTTTTCATCG	pBrxXL$_{Sty}$ PARIS Gibson assembly REV (1)
TRB1740	CGATGAAAACGTTTCAGTTTGCTCATGGAAAACGGTGTAA	pBrxXL$_{Sty}$ PARIS Gibson assembly FWD (2)
TRB1741	TTCTTCGTTGGTCAGAAACACGTATTTGTCTTCAACACGT	pBrxXL$_{Sty}$ PARIS Gibson assembly REV (2)
TRB1742	ACGTGTTGAAGACAAATACGTGTTTCTGACCAACGAAGAA	pBrxXL$_{Sty}$ PARIS Gibson assembly FWD (3)
TRB1743	AATCCCATTTCATTCCAGATTTAAATAATCTCCGGTGCAT	pBrxXL$_{Sty}$ PARIS Gibson assembly KO REV (3)
TRB1788	GATGAAAACGTTTCAGTTTGCTCATGGAAAACGGTGTAACA	pBrxXL$_{Sty}$ *brxC* Gibson assembly KO FWD (1)
TRB1790	GAATCCGGTCACCTGCCTCAGCTGTGCTCTTTAACGAGGA	pBrxXL$_{Sty}$ *brxC* Gibson assembly KO REV (1)
TRB1791	TCCTCGTTAAAGAGCACAGCTGAGGCAGGTGACCGGATTC	pBrxXL$_{Sty}$ *brxC* Gibson assembly KO FWD (2)
TRB1792	CCGGCGCGATCAGATAACTTTCAATACAAAAACGCGGAAGAACC	pBrxXL$_{Sty}$ *brxC* Gibson assembly KO REV (2)
TRB1793	CTTCCGCGTTTTTGTATTGAAAGTTATCTGATCGCGCCGG	pBrxXL$_{Sty}$ *brxC* Gibson assembly KO FWD (3)
TRB1794	GTTACACCGTTTTCCATGAGCAAACTGAAACGTTTTCATCGCT	pBrxXL$_{Sty}$ *brxC* Gibson assembly KO REV (3)
TRB1778	GATGAAAACGTTTCAGTTTGCTCATGGAAAACGGTGTAAC	pBrxXL$_{Sty}$ *ariA* Gibson assembly KO FWD (1)
TRB1795	AGTGTTTTCAGTAAGTTGCCATCGAACTCATCAAGAATGC	pBrxXL$_{Sty}$ *ariA* Gibson assembly KO REV (1)
TRB1796	GCATTCTTGATGAGTTCGATGGCAACTTACTGAAAACACT	pBrxXL$_{Sty}$ *ariA* Gibson assembly KO FWD (2)
TRB1797	TTGCCATCGGTTAAATCAATTTAAATAATCTCCGGTGCAT	pBrxXL$_{Sty}$ *ariA* Gibson assembly KO REV (2)
TRB1798	ATGCACCGGAGATTATTTAAATTGATTTAACCGATGGCAA	pBrxXL$_{Sty}$ *ariA* Gibson assembly KO FWD (3)
TRB1799	GTTACACCGTTTTCCATGAGCAAACTGAAACGTTTTCATC	pBrxXL$_{Sty}$ *ariA* Gibson assembly KO REV (3)

Primers used for LIC cloning into pBAD30-LIC

TRB1587	caacagcagacgggaggtAATACCAATAACATCAAAAA	FWD LIC PglX Salmonella D23580
TRB1588	gcgagaaccaaggaaaggttattaAATAATCTCCGGTGCATTGC	REV LIC PglX Salmonella D23580
TRB1581	caacagcagacgggaggtATCGATCCCGTGCTTGAATA	FWD LIC BrxB Salmonella D23580
TRB1582	gcgagaaccaaggaaaggttattaACGAGGATTCAATGTCGCCG	REV LIC BrxB Salmonella D23580
TRB1589	caacagcagacgggaggtACCGACCAGTCGCAGCTGGC	FWD LIC PglZ Salmonella D23580
TRB1590	gcgagaaccaaggaaaggttattaAAAGAAATCATCCTGAAATG	REV LIC PglZ Salmonella D23580
TRB1856	tggagccacccgcagttcgaaaaaTCAGGAGTCAAGACTGAG	FWD for cloning Strep site into pSAT1-LIC
TRB1857	tttttcgaactgcgggtggctccaCGGATGATGATGATGATGATG	REV for cloning Strep site into pSAT1-LIC
TRB2025	CAACAGCAGACGGGAGGTCCTCTAGAAATAATTTTGTTTAAC	FWD LIC pBAD30-RBS-His-Strep-PglX
TRB2026	GCGAGAACCAAGGAAAGGTTATTAGTTATTAAATAATCTCCGGTG	REV LIC pBAD30-RBS-His-Strep-PglX
TRB2027	CAACAGCAGACGGGAGGT GAAGGAGATATATCCATG AATACCAATAACATCAAAAAATAC	FWD LIC pBAD30-RBS-PglX
TRB2028	GCGAGAACCAAGGAAAGGTTATTA TTATTAAATAATCTCCGGTGC	REV LIC pBAD30-RBS-PglX

Primers used to make D23580 *brxL* KO

AB-110	TGTGATTATCGATCTGGCATTTCAGGATGATTTCTTTT AAGTGAGGCGCTTGTGTAGGCTGGAGCTGCTTC	FWD Deletion of the *brxL* gene in *S*. Typhimurium D23580 ΔΦ by λ red recombination
AB-111	TAACGACGATATTGATTTTATCGAGGAGTTTG ACCCACTCCTCGATATATCATATGAATATCCTCCTTAG	REV Deletion of the *brxL* gene in *S*. Typhimurium D23580 ΔΦ by λ red recombination
AB-112	CTGGCTGATACTGGAAGATGCAC	FWD External primer to check *brxL* deletion in *S*. Typhimurium D23580 ΔΦ
AB-113	GACGATTTAGTACGTGCGTTAGTGG	REV External primer to check *brxL* deletion in *S*. Typhimurium D23580 ΔΦ

Sequencing primers (ranges indicate position of primer in *Salmonella* BREX coding region)

TRB710	CGTTACCTGGAACCATTCGT	2289-2308
TRB711	CCCTATGGATAGCTGGGATG	3002-3021
TRB712	GCAGGACGTGATGGGTTTTA	3688-3707
TRB713	GCCAATACGACGCGTTTAAG	4403-4402
TRB714	GTCTATCCGGACCAAAGGTG	5094-5113
TRB715	CGGCTGCATTTTAATTCGTT	5796-5815
TRB716	GCACAAACTATGGCGGAAAT	6499-6518
TRB717	ACGGATGCCGAGAAGAAGAT	7195-7214
TRB718	GATAACCCGACAGGCTTTGA	7888-7907
TRB719	GTCTCGACATTGACGACCG	8600-8618
TRB720	ATTACGATGGCACATTTGGG	9292-9311
TRB721	GGATTAATGTGCACTCCGGT	10000-10020
TRB722	AAATCTCGAATTTATCGCCG	10704-10723
TRB723	ATTGGCTGGGCACGGGTA	11398-11415
TRB724	GGCGGTGTTGTACTCATTGAT	12105-12126
TRB725	CCGGTTTTAATACTGCGTTTC	12788-12808
TRB726	CTTGAAAGGCCTGGTCACTG	13505-13524
TRB727	TTGCTGAATCTGCGTAATCG	14193-14212
TRB728	GCATAACACCATTGATGCCA	14888-14907
TRB729	CGAATTTCAATCGCCGTAAT	15598-15618
TRB730	GTATTTACCGCACGTACGCA	FOR_21
TRB731	AACCAGCGCGACGTTATC	FOR_22
TRB732	CGCGGTAGATATTCCGACTG	FOR_23
TRB733	CAACCTCATCCTCTTCACCTG	FOR_24

Probes for mass photometry and analytical size exclusion chromatography

TRB2180	GGTATCCGTTCGTCATAGGCTCAAAGCGTCtcctgagtctcgG ATCAGtacgaactaccggaaccaacacgaCTGATCgtcttcggtgacC ATTGCAACACTAGTATCTCGAGTGCCGTG	dsDNA oligo for Refeyn/A-SEC FWD1.2
TRB2181	CAC GGC ACT CGA GAT ACT AGT GTT GCA ATG GTC ACC GAA GAC GAT CAG TCG TGT TGG TTC CGG TAG TTC GTA CTG ATC CGA GAC TCA GGA GAC GCT TTG AGC CTA TGA CGA ACG GAT ACC	dsDNA oligo for Refeyn/A-SEC REV1.2
TRB2182	GGTATCCGTTCGTCATAGGCTCAAAGCGTCtcctgagtctcgG ATCAGtacgaactaccggaaccaacacgaGATCAGgtcttcggtgacC ATTGCAACACTAGTATCTCGAGTGCCGTG	dsDNA oligo for Refeyn/A-SEC FWD2.2
TRB2183	CAC GGC ACT CGA GAT ACT AGT GTT GCA ATG GTC ACC GAA GAC CTG ATC TCG TGT TGG TTC CGG TAG TTC GTA	dsDNA oligo for Refeyn/A-SEC REV2.2

	CTG ATC CGA GAC TCA GGA GAC GCT TTG AGC CTA TGA CGA ACG GAT ACC	
TRB2184	GGTATCCGTTCGTCATAGGCTCAAAGCGTCtcctgagtctcgG ATCAGtacgaactaccggaaccaacacgacaatacgtcttcggtgacCAT TGCAACACTAGTATCTCGAGTGCCGTG	dsDNA oligo for Refeyn/A-SEC FWD3.2
TRB2185	CAC GGC ACT CGA GAT ACT AGT GTT GCA ATG GTC ACC GAA GAC GTA TTG TCG TGT TGG TTC CGG TAG TTC GTA CTG ATC CGA GAC TCA GGA GAC GCT TTG AGC CTA TGA CGA ACG GAT ACC	dsDNA oligo for Refeyn/A-SEC REV3.2
TRB2186	GGTATCCGTTCGTCATAGGCTCAAAGCGTCtcctgagtctcgtgt acctacgaactaccggaaccaacacgacaatacgtcttcggtgacCATTG CAACACTAGTATCTCGAGTGCCGTG	dsDNA oligo for Refeyn/A-SEC FWD4.2
TRB2187	CAC GGC ACT CGA GAT ACT AGT GTT GCA ATG GTC ACC GAA GAC GTA TTG TCG TGT TGG TTC CGG TAG TTC GTA GGT ACA CGA GAC TCA GGA GAC GCT TTG AGC CTA TGA CGA ACG GAT ACC	dsDNA oligo for Refeyn/A-SEC REV4.2

Supplementary Table 4. Plasmids used in this study.

Plasmid	Notes	Primers used	Reference
pBrxXL$_{Sty}$	Full *S. enterica* serovar Typhimurium coding region in a pGGA vector backbone, created by golden gate assembly from genomic *S. enterica* serovar Typhimurium D23580 DNA.	TRB1367-TRB1378	This study
pBrxXL$_{Sty}$-Δ*brxA*	Cloned by Genscript from pBrxXL$_{Sty}$.	Genscript synthesis	This study (Genscript)
pBrxXL$_{Sty}$-Δ*brxB*	Cloned by Genscript from pBrxXL$_{Sty.}$	Genscript synthesis	This study, (Genscript)
pBrxXL$_{Sty}$-Δ*brxC*	Created by Gibson assembly from pBrxXL$_{Sty.}$	TRB1788, TRB1790-TRB1794	This study
pBrxXL$_{Sty}$-Δ*pglX*	Created by Gibson assembly from pBrxXL$_{Sty.}$	TRB1734-TRB1737	This study
pBrxXL$_{Sty}$-Δ*pglZ*	Cloned by Genscript from pBrxXL$_{Sty.}$	TRB904/907	This study, (Genscript)
pBrxXL$_{Sty}$-Δ*brxL*	Cloned by Genscript from pBrxXL$_{Sty.}$	TRB904/958	This study
pBrxXL$_{Sty}$-Δ*ariA*	Created by Gibson assembly from pBrxXL$_{Sty.}$	TRB1778, TRB1795-TRB1799	This study
pBrxXL$_{Sty}$-Δ*ariB*	Cloned by Genscript from pBrxXL$_{Sty.}$	Genscript synthesis	This study, (Genscript)
pBrxXL$_{Sty}$-Δ*ariA*Δ*ariB*	Created by Gibson assembly from pBrxXL$_{Sty.}$	TRB1748-TRB1743	This study
pTRB507	pGGA plasmid backbone containing 12400 – 14394 of the pEFER plasmid from *E. fergusonii,* used as a negative control.		Picton *et al.* 2021
pSAT1-LIC	pBAT4 derivative; pMB1 replicon		Cai *et al.* 2020
pSAT1-Strep-6xHis-SUMO	Created by Gibson Assembly	TRB1856 TRB1857	This study
pSAT1-6xHis-SUMO-pglX	Created by LIC	TRB1587 TRB1588	This study
pBAD30-Strep-6xHis-SUMO-pglX	Created by LIC	TRB2025 TRB2026	This study
pBAD30-Strep-6xHis-SUMO-pglX NTD	Cloned by Genscript. NTD of PglX only.	Genscript synthesis	This study, (Genscript)
pBAD30-Strep-6xHis-SUMO-pglX^{Mutant}	Cloned by Genscript. Contains K616A, K1201A, K1097A, D1213A, K1110A and K1070A	Genscript synthesis	This study, (Genscript)
pBAD30-pglX	Created by GA	TRB2027 TRB2028	This study
pSAT1-6xHis-SUMO-brxB	Created by LIC	TRB1581 TRB1582	This study
pSAT1-6xHis-SUMO-pglZ	Created by LIC	TRB1589 TRB1590	This study
pBAD30-ocr	Created by LIC	TRB1911 TRB2081	This study

pBAD30-*pglX*(mut.1–23)	Derived from pBAD30-*pglX.* Twenty-three plasmids each with different mutant *pglX* genes, described in Table S3.	Genscript synthesis	This study
